# Can functional walk tests add value to the prediction of cardiorespiratory fitness after stroke? A prospective cohort study

**DOI:** 10.1371/journal.pone.0255308

**Published:** 2021-08-02

**Authors:** Mari Gunnes, Inger-Lise Aamot Aksetøy, Turid Follestad, Bent Indredavik, Torunn Askim

**Affiliations:** 1 Department of Neuromedicine and Movement Science, Faculty of Medicine and Health Sciences, Norwegian University of Science and Technology, Trondheim, Norway; 2 Stroke Unit, Department of Internal Medicine, St. Olav’s University Hospital, Trondheim, Norway; 3 Department of Circulation and Medical Imaging, Faculty of Medicine and Health Sciences, Norwegian University of Science and Technology, Trondheim, Norway; 4 Department of Clinical and Molecular Medicine, Faculty of Medicine and Health Sciences, Norwegian University of Science and Technology, Trondheim, Norway; Prince Sattam Bin Abdulaziz University, College of Applied Medical Sciences, SAUDI ARABIA

## Abstract

**Background:**

Cardiorespiratory fitness is often impaired following stroke, and peak oxygen consumption (VO_2peak_) is an important prognostic value of all-cause mortality. The primary objective was to investigate whether functional walk tests assessed in the subacute phase after stroke added value in predicting VO_2peak_ in chronic stroke, in addition to age, sex and functional dependency. Secondary objectives were to investigate associations between daily physical activity and functional walk tests, and with VO_2peak_ in chronic stroke.

**Methods:**

This prospective cohort study included eligible participants originally included in the randomized controlled trial Life After Stroke. Functional walk tests, i.e., six-minute walk test (6MWT) and maximal gait speed, were assessed at inclusion and 18 months later. VO_2peak_ [ml/kg/min] was assessed by a cardiopulmonary exercise test on a treadmill 20 months after inclusion. Daily physical activity was measured by a uniaxial accelerometer (activPAL) at 18-month follow-up.

**Results:**

Ninety-two community-dwelling individuals, with a mean (SD) age of 69.2 (10.6) years and 33 (35.9%) women, were included 3 months after stroke onset. Eighty-three (90.2%) participants had a modified Rankin Scale (mRS) score of 1 or 2, indicating functional independence. An overall assessment of four prediction models indicated the combination of age, sex, mRS and 6MWT as predictors to be the best fitted model in predicting VO_2peak_ (adjusted R^2^ = 0.612). Secondary results showed statistically significant, but not clinically significant, associations between daily physical activity and functional walk tests, and with VO_2peak._

**Conclusions:**

6MWT add significant value to the prediction of mean VO_2peak_ in the chronic phase in mild strokes, in combination with age, sex and functional dependency. This prediction model may facilitate clinical decisions and rehabilitation strategies for mildly affected stroke survivors in risk of low levels of VO_2peak_. Future studies should validate the model in various stages after stroke and in patients moderately and severely affected.

## Introduction

Cardiorespiratory fitness (CRF), measured as peak oxygen consumption (VO_2peak_), is often impaired following stroke. VO_2peak_ assessed throughout all stages post-stroke are shown to range between 26% and 87% of normative values [1]. A strong inverse relationship between CRF and all-cause mortality in healthy individuals [2] and patients with cardiovascular and other chronic diseases [3, 4] has established its prognostic value. In individuals after stroke, low CRF often leads to low social participation and reduced quality of life [5]. In addition, it exacerbates underlying cardiovascular and metabolic risk factors, which, in turn, contribute to increased risk of recurrent stroke [6].

Despite the well-established importance of CRF, it is currently the only major risk factor that is not routinely or regularly assessed after stroke [7, 8]. A cardiopulmonary exercise test (CPET) is considered the gold standard for measuring VO_2peak_ [9]. However, a CPET is expensive, time-consuming, and requires trained staff and advanced laboratory equipment often unavailable in rehabilitation facilities. VO_2peak_ is, however, closely related to several factors associated with health status, including age, sex and functional disability after stroke [10]. Despite some inconsistency due to methodological variability, previous cross-sectional studies have also shown that functional walk tests assessing walking capacity are associated with VO_2peak_ post-stroke [11]. To address whether functional walk tests add value to a prediction model including already established determinants of CRF, like age, sex and functional disability, would be useful for determining the possibility of estimating VO_2peak_ without requiring CPET.

Further, improved walking capacity and increased CRF are important rehabilitation targets following stroke. These are also commonly used outcome measures in rehabilitation studies aiming to increase daily physical activity. However, previous research has yielded inconsistent findings regarding the relationship between daily physical activity and functional walk tests [12–15], and between daily physical activity and VO_2peak_ [6, 15–17] after stroke. Further investigations confirming how walking capacity and CRF is associated with daily physical activity in community-dwelling individuals after stroke would be useful in order to optimize future post stroke rehabilitation.

The study objectives were to test the hypotheses that (a) walking capacity, as measured by six-minute walk test (6MWT) and maximal gait speed, in the subacute phase after stroke, add value to the prediction of VO_2peak_ in the chronic phase, in addition to age, sex and functional disability, and that (b) higher levels of walking capacity and CRF, respectively, are associated with higher levels of daily physical activity in chronic stroke.

## Materials and methods

### Study design, setting, and participants

This prospective, observational cohort study represents extended analyses of a subsample of participants originally included in the multisite, randomized controlled clinical trial Life After Stroke (LAST) [18]. No statistically significant differences were shown between the intervention and control group in LAST, hence, participants in the present study were treated as one group.

For LAST, participants were recruited from October 18, 2011 to June 26, 2014 at the outpatient clinics of the stroke units of two Norwegian hospitals and consecutively randomized 10 to 16 weeks after acute stroke. Eligible individuals met each of the following inclusion criteria: diagnosed with first-ever or recurrent stroke (infarction or intracerebral hemorrhage), age ≥ 18 years, discharged from hospital or inpatient rehabilitation at inclusion, community dwelling, modified Rankin Scale (mRS) score < 5, and cognitive function as evaluated by the Mini-Mental State Examination (MMSE) > 20 points (>16 points for participants with aphasia). Exclusion criteria were serious medical comorbidity with shortened life expectancy or a condition contraindicating motor training. Informed, consenting participants were randomly allocated to either the intervention group or the control group and followed prospectively for 18 months after inclusion [19].

At 18-month follow-up, participants assigned to one of the sites (i.e., Trondheim) were invited to participate in the present study. Cardiopulmonary exercise tests (CPETs) were performed within 3 months after the 18-month follow-up, i.e., approximately 20 months after inclusion. Only individuals considered able to tolerate the CPET were included, and a medical assessment was performed to screen for any comorbidity that might represent contraindications. Eligible participants were able to walk (with or without an assistive device but without personal assistance) and understand simple oral instructions in order to perform the test. Participants were excluded if they suffered advanced congestive heart failure, peripheral arterial disease with claudication, unstable angina, uncontrolled hypertension, severe cognitive impairments or aphasia, and/or significant orthopedic or pain conditions that limited participation.

All participants gave written informed consent. Ethical approval for the study was granted by the Regional Committee of Medical and Health Research Ethics (REC no. 2011/1427) and registered with ClinicalTrials.gov (no. NCT01467206).

### Baseline assessments

At inclusion, age, sex, living condition, type of stroke, and medical history were recorded. Stroke severity was measured by the National Institutes of Health Stroke Scale (NIHSS) [20], which is a neurologic scale that assesses the severity of neurological impairments of stroke; a high overall score indicates severe symptoms [20]. The modified Rankin Scale (mRS) was used to assess the overall level of functional independence [21]. mRS is a global outcome measure that combines physical, mental, and speech aspects into a single score graded as 0 (no symptoms at all), 1 (no significant disability), 2 (slight disability), 3 (moderate disability), 4 (moderately severe disability), 5 (severe disability) or 6 (deceased) [21]. Cognitive function was assessed by the Mini-Mental State Examination (MMSE) [22], which is a brief screening tool that provides a quantitative assessment of cognitive impairment. A summary score between 0 and 30 (maximal) of 11 questions or tasks assess different cognitive domains, such as orientation to time and place, attention, calculations, language, and visual constructions [22].

### Outcome measurements

#### Cardiopulmonary exercise test (CPET)

CRF level was defined by VO_2peak_ [ml/kg/min] and assessed by a symptom limited CPET obtained by a treadmill protocol. As deconditioned or elderly individuals often cannot meet the rigorous conditions for maximal oxygen consumption (VO_2max_), assessing exercise capacity by VO_2peak_ is more commonly used [9, 23].

After a 10-minute warm-up (i.e., treadmill walking without incline, used to assess gait safety and to select target walking velocity), an individualized ramp protocol was used. Participants were encouraged not to use the handrails, but minimal handrail support was allowed to keep their balance if necessary. Participants had to walk at their fastest preferred walking speed, and the workload was increased every minute by increasing the inclination by 2–3%. The test was terminated by standard clinical criteria according to guidelines by the American College of Cardiology [24]. Ventilatory gas measurements were performed with a MetaMax II (Cortex Biophysics, Germany), using mixing chamber analysis with sample frequency of 10 seconds. The highest average over 30 seconds was determined as VO_2peak_. The MetaMax was calibrated every test day using the standard two-point gas calibration procedure, including measurements of ambient air and a gas mix (16% O_2_ and 4% CO_2_), a calibration of the volume transducer with a calibrated 3 L syringe and barometric pressure, as recommended by the manufacturer [25].

For safety reasons, the blood pressure (BP) was measured at rest (before the test procedure), directly after participants reached their maximal exercise level during CPET, and 5–10 minutes post-test to confirm an approximate return of BP to baseline. An OSZ5 automated blood pressure monitor (Welch Allyn, Germany) was used. Further, heart function was monitored continuously with a 12-lead electrocardiogram during CPET, and testing was discontinued if the subject showed any sign of cardiovascular dysfunction. The test was performed in a location with continuous access to medical assistance and a short distance to the emergency unit.

#### Functional walk tests

For 6MWT, participants were requested to ‘walk as far as possible for six minutes, but no jogging or running’ on a 30-meter-long course according to standard protocol; farther distance walked indicated higher walking endurance [26]. During the test, participants could use their usual walking aids. They were allowed to slow down, stop, or rest as necessary, and resume walking as soon as they were able to. Further, the standardized phrases for encouragement were used every minute, as specified in the guidelines of the American Thoracic Society [27].

Maximal gait speed was measured over a 10-meter distance, with two meters at each end for acceleration/deceleration [28]. The participants were instructed to walk as fast as they could safely without running. The procedure was performed twice at both test occasions, whereas the fastest attempt of the two tests at each occasion were used for further analyses.

#### Daily physical activity

Daily physical activity was measured by a uniaxial accelerometer (activPAL, PAL Technologies Ltd, Glasgow, UK), which was attached to the participants’ unaffected thigh and worn for at least 4 consecutive days at 18-month follow-up. It has been shown that the accelerometer accurately determines the amount of time spent upright during standing and walking activities [29]. Hence, in the present study, daily physical activity was defined as the average number of hours spent walking between 07.00 am and 11.00 pm each day. The activPAL software package (activPAL Professional Research Edition) was used to process the raw acceleration-data signals from the accelerometers. The average daily time spent walking was calculated for each participant. The daily physical activity was recorded as missing if a participant had validly recorded data for less than 2 days.

### Statistical analyses

Baseline characteristics at inclusion were compared between included and non-included participants (i.e., those screened but ineligible due to prescribed criteria) using independent samples t-tests, Chi-squared tests with continuity corrections, or Fisher’s exact tests as appropriate. The distribution of continuous variables and residuals from linear regressions was visually inspected by histograms and normal quantile-quantile (Q-Q) plots. Multiple ordinary least-squares linear regression was used to assess the added value of the two functional walk tests to predict VO_2peak,_ compared to a model that included only age, sex and mRS (model A). All included predictors were assessed at inclusion. Three multiple regression models were considered, including either 6MWT [m] (model B), maximal gait speed [m/s] (model C), or both in combination (model D), as additional predictors to model A. Further, an interaction term between age and sex was included in each model. The models were checked for violation of the assumptions of multicollinearity, normality, linearity, and homoscedasticity. The model fit was compared between the models with model A defined as the reference model, by likelihood ratio tests, the Akaike information criterion (AIC) and the Bayesian information criterion (BIC). To study the out-of-sample performance, 5-fold cross-validation was used.

Multiple linear regressions were also used to assess the associations between walking [hours/day] as dependent variable, and 6MWT [m], maximal gait speed [m/s], and VO_2peak_ [ml/kg/min] as predictors in three separate regression models, each adjusted for age, sex and mRS. Walking was assessed at 18-month follow-up, and contrary to our primary analyses, results from 6MWT, maximal gait speed, and covariates were also assessed 18 months after inclusion for these secondary analyses.

Two-sided P-values < .05 were considered statistically significant, and results from multiple linear regressions were reported as unstandardized regression coefficients with 95% confidence intervals (CI) and p-values. The statistical analyses were performed using SPSS 25.0, Stata/MP 16, and Excel 2016.

## Results

**Fig 1:**
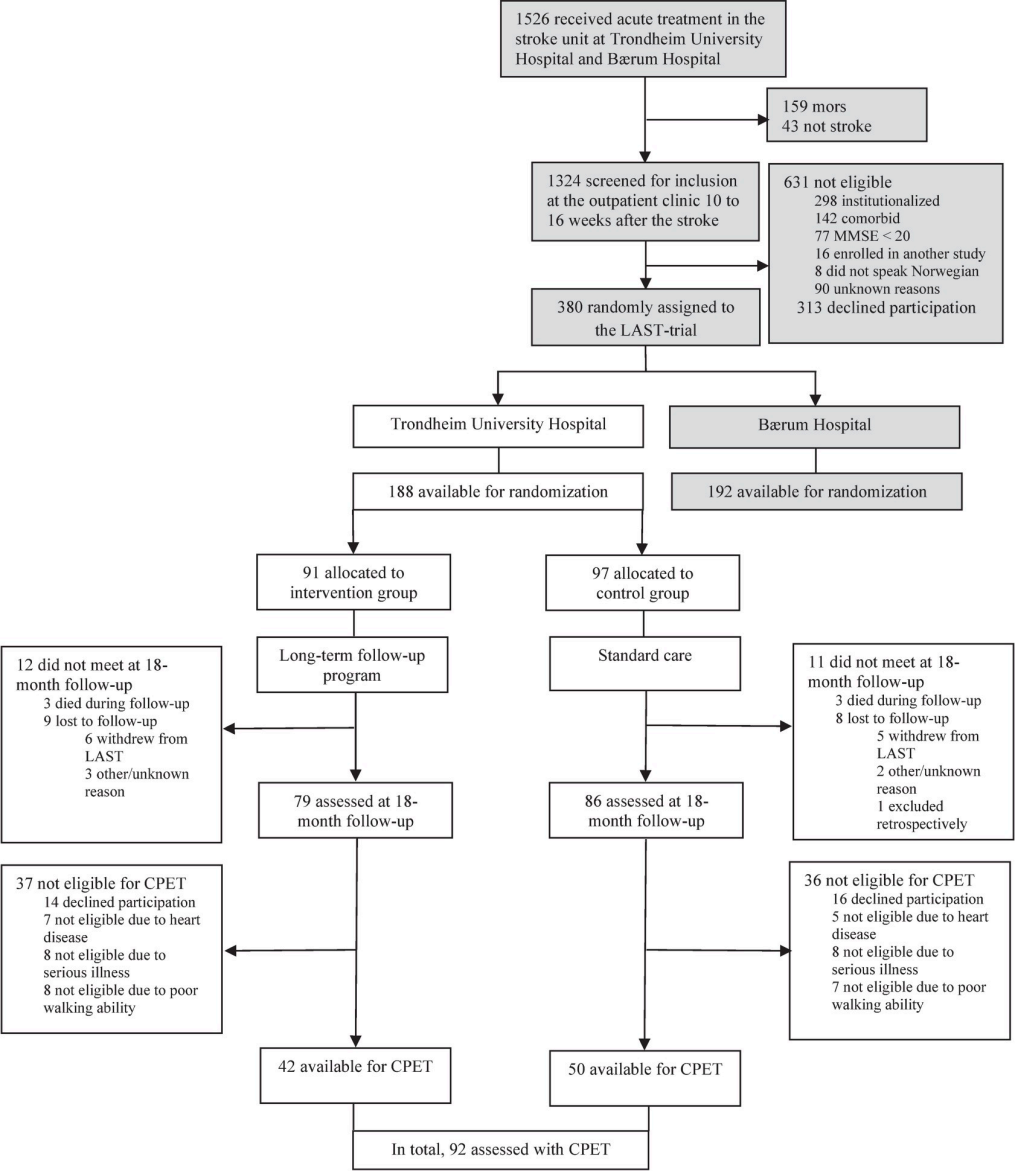
Flowchart of study participants and reasons for non-inclusion in the study. Abbreviations: MMSE, Mini-Mental State Examination; LAST, Life After Stroke; CPET, cardiopulmonary exercise test.

Of the 380 participants enrolled in LAST, 188 (49.5%) were assigned to St. Olav’s University Hospital. For different reasons specified in the flowchart (Fig 1), 23 (12.2%) participants were not retested in LAST 18 months later, leaving 165 (87.8%) subjects assessed at follow-up. Among those, 73 (44.2%) participants were not eligible for CPET, leaving a total of 92 participants eligible for the study, with test procedures performed from May 2013–January 2016.

The study sample had a mean (SD) age of 69.2 (10.6) years at baseline and included 33 (35.9%) women and 59 (64.1%) men (Table 1). Most participants scored 1 (n = 61, 66.3%) or 2 (n = 22, 23.9%) at the mRS.

**Table 1:** Baseline demographics and stroke characteristics of participants.

		Total sample (n = 92)	Non-participants (n = 96)	P
**Demographics**				
Age, mean (SD)		69.2 (10.6)	73.8 (10.62)	0.003*
	< 80 y	78 (84.8)	67 (69.8)	0.023†
	≥ 80 y	14 (15.2)	29 (30.2)	
Sex				
	Female	33 (35.9)	47 (49.0)	0.096†
	Male	59 (64.1)	49 (51.0)	
Domestic circumstances				
	Living with someone	75 (81.5)	59 (61.5)	0.004†
	Living alone	17 (18.5)	37 (38.5)	
MMSE score, mean (SD)		28.03 (2.26)	28.03 (2.28)	0.997*
	≥25	83 (90.2)	84 (87.5)	0.700†
	<25	8 (8.7)	11 (11.5)	
**Stroke characteristics**				
Days after stroke, mean (SD)		105.9 (12.9)	104.7 (13.5)	0.535*
Stroke type				
	Infarction	83 (90.2)	94 (97.9)	0.053†
	Hemorrhage	9 (9.8)	2 (2.1)	
NIHSS score, mean (SD)		1.17 (1.45)	2.21 (2.73)	0.001*
Range		0 to 7	0 to 12	
Mild stroke	<8	92 (100)	91 (94.8)	
Moderate stroke	8–16	0	5 (5.2)	
Severe stroke	>16	0	0	
mRS score, mean (SD)		1.43 (0.67)	2.08 (0.94)	<0.001*
	mRS = 1	61 (66.3)	32 (33.4)	
	mRS = 2	22 (23.9)	30 (31.3)	
	mRS = 3	9 (9.8)	28 (29.2)	
	mRS = 4	0	6 (6.3)	
Comorbidity, prior to stroke onset				
	Stroke	12 (13.0)	28 (29.2)	0.012†
	TIA	14 (15.2)	12 (12.5)	0.743†
	Myocardial infarction	7 (7.6)	25 (26.0)	0.002†
	Heart failure	0	5 (5.2)	0.059‡
	Atrial fibrillation	12 (13.0)	31 (32.3)	0.003†
	Hypertension	50 (54.3)	68 (70.8)	0.029†
	Diabetes	9 (9.8)	20 (20.8)	0.058†
	Lung diseases	12 (13.0)	21 (21.9)	0.162†

Data are shown for both participants included in the study (n = 92) and for non-participants (n = 96), i.e., those screened but not included in the study due to exclusion criteria. P-values show group differences between total sample and non-participants. Abbreviations: MMSE, Mini-Mental State Examination; NIHSS, National Institutes of Health Stroke Scale; mRS, modified Rankin Scale; SD, standard deviation; P, P-value; TIA, transient ischemic attack. Data are reported as numbers (percentages) of participants unless otherwise indicated.

* Independent samples t-test

† Chi-Square test with continuity correction

‡ Fisher’s exact test

Results from the CPET, activPAL and functional walk tests at inclusion and follow-up are presented in Table 2. All individuals remained asymptomatic during CPET, normal blood pressure responses were observed, and no adverse events were recorded. Among the total sample, 2 individuals declined to wear the accelerometer and data from 7 participants were missing due to technical problems with the sensor. Hence, a total of 83 participants (31 women and 52 men) provided valid accelerometry data, whereas 7 individuals wore the activPAL for 3 days and 2 persons for 2 days instead of 4 days as prescribed by the protocol. During the functional walk tests, five individuals used a cane/crutch at inclusion, and one of them used a cane/crutch at the 18-month follow-up. During quality check of the functional walk tests, one measurement error was deleted from the related analyses among the maximal gait speed data at inclusion.

**Table 2:** Results from the functional walk tests, daily physical activity as measured with activPAL, and cardiopulmonary exercise tests.

		Women		Men		Total	
		Inclusion	Follow-up*	Inclusion	Follow-up*	Inclusion	Follow-up*
**Functional walk tests**							
6MWT [m]		429.8 (102.0)	446.0 (95.2)	546.9 (128.5)	534.0 (128.7)	504.9 (131.8)	502.4 (124.7)
	Range	259.0 to 656.0	243.0 to 619.0	250.0 to 860.0	215.0 to 813.0	250.0 to 860.0	215.0 to 813.0
Maximal gait speed [m/s]		1.65 (0.38)	1.58 (0.38)	2.12 (0.48)	1.99 (0.57)	1.95 (0.50)	1.84 (0.54)
	Range	0.85 to 2.41	0.73 to 2.28	0.94 to 2.98	0.91 to 3.51	0.85 to 2.98	0.73 to 3.51
**activPAL**							
Walking [hours/day]		-	1.49 (0.60)	-	1.63 (0.74)	-	1.58 (0.69)
	Range	-	0.34 to 2.83	-	0.29 to 3.80	-	0.29 to 3.80
**Cardiopulmonary exercise test (CPET)**							
VO_2peak_ [ml/kg/min]		-	22.31 (4.99)	-	28.57 (8.91)	-	26.32 (8.27)
	Range	-	13.75 to 39.50	-	13.86 to 54.44	-	13.75 to 54.44
VO_2peak_ [l/min]		-	1.55 (0.46)	-	2.36 (0.84)	-	2.07 (0.82)
	Range	-	0.79 to 2.84	-	1.03 to 4.46	-	0.79 to 4.46
RER_peak_		-	1.07 (0.10)	-	1.07 (0.10)	-	1.07 (0.10)
	Range	-	0.86 to 1.24	-	0.87 to 1.34		0.86 to 1.34
HR_peak_		-	146 (18)	-	150 (21)	-	148 (20)
	Range	-	109 to 169	-	103 to 196		103 to 196
Blood pressure, pre CPET							
	Systolic	-	148.8 (19.6)	-	148.4 (20.5)	-	148.6 (20.1)
	Diastolic	-	88.5 (11.3)	-	90.1 (10.3)	-	89.5 (10.6)
Height [cm]		-	163.5 (5.0)	-	178.6 (5.9)	-	173.2 (9.2)
Weight [kg]		-	70.0 (15.5)	-	82.4 (15.2)	-	78.0 (16.4)
BMI		-	26.1 (5.2)	-	25.8 (4.1)	-	25.9 (4.5)

Data from a total of 92 participants, divided by women (n = 33) and men (n = 59), are presented at inclusion and follow-up. However, data from maximal gait speed at inclusion was n = 91 due to 1 omitted outlier (measurement error). Among the participants with eligible data from activPAL, n = 83, divided by 31 women and 52 men. Abbreviations: 6MWT, six-minute walk test; VO_2peak,_ peak oxygen consumption; RER_peak,_ peak respiratory exchange ratio; HR_peak,_ peak heart rate; CPET, cardiopulmonary exercise test; BMI, body mass index. Data are presented as mean (SD) values unless otherwise stated. Range indicate min-max, and dash (-) indicate data not applicable.

*Follow-up was 18 months after inclusion for 6MWT, gait speed and activPAL, and 20 months after inclusion for CPET.

**Fig 2:**
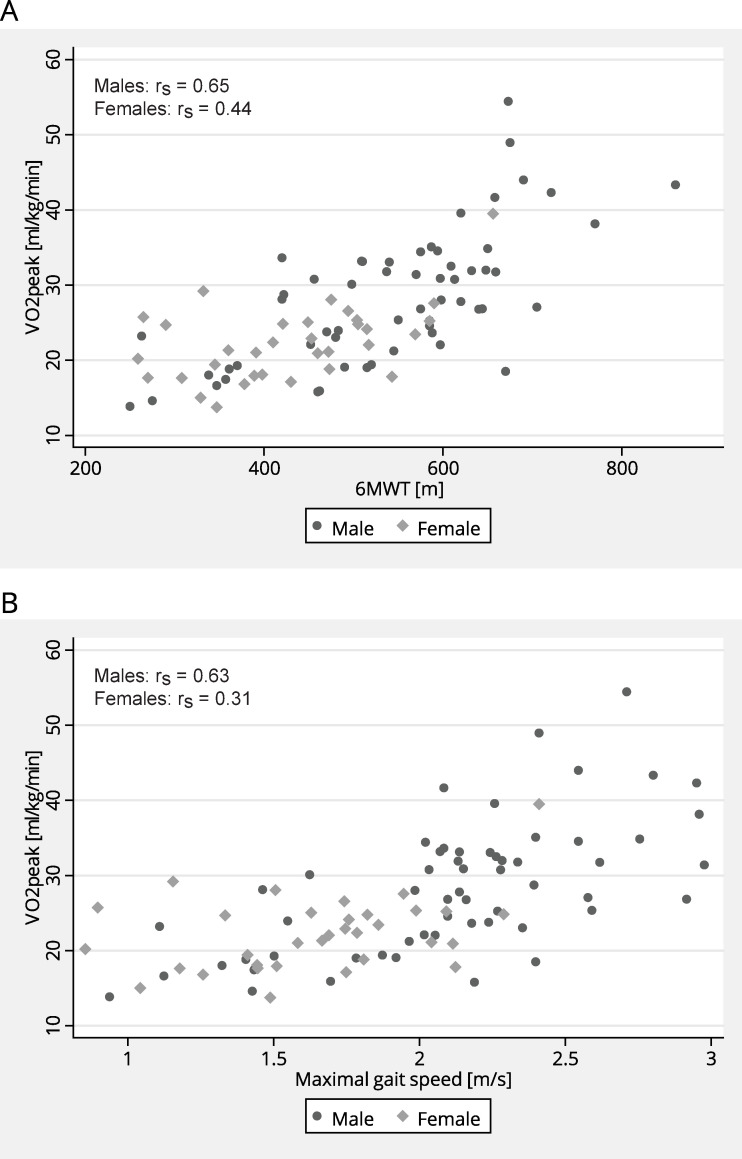
Relationship between cardiorespiratory fitness and functional walk tests. Scatterplots illustrating the relationship between VO_2peak_ [ml/kg/min] in chronic stroke and (A) 6-minute walk test (6MWT) [m], and (B) maximal gait speed [m/s] in subacute phase after stroke. Abbreviation: r_s_ = Spearman correlation coefficient.

Relationships between VO_2peak_ and the functional walk tests at inclusion are shown for males and females separately in Fig 2. No interaction effects between sex and the functional walk tests were found to be statistically significant when included in the regression models. Hence, results from models without these interactions are presented. The multiple regression analyses showed that age, sex, and mRS alone (model A) accounted for 52.2% of the variance in VO_2peak_ (Table 3). After entry of the functional walk tests, both separately and in combination, the adjusted R^2^ increased from 49.4% (model A) to 61.2% (model B), 56.7% (model C) and 61.3% (model D), respectively (Table 3). Results from the likelihood ratio tests, AIC, and BIC, in addition to results from the cross-validation (i.e., cross-validated R^2^ and RMSE), indicated that model B should be considered the best fitted model.

**Table 3:** Comparison of the models A, B, C and D. Models are based on data from a total of 91 participants.

Model	R^2^	Adjusted R^2^	Change in adjusted R^2^ from model A	RMSE	AIC	BIC	P-value, LR-test vs model A	R^2^ cross validated	RMSE Cross validated
**A:** Age, sex, age*sex, mRS	0.522	0.494	-	5.793	583.76	598.82	-	0.521	6.123
**B:** Age, sex, age*sex, mRS, 6MWT	0.638	0.612	0.118	5.072	560.57	578.04	<0.001	0.615	5.228
**C:** Age, sex, age*sex, mRS, max gait speed	0.596	0.567	0.073	5.356	570.41	587.99	0.001	0.572	5.553
**D:** Age, sex, age*sex, mRS, 6MWT, max gait speed	0.643	0.613	0.119	5.067	561.23	581.31	< 0.001	0.601	5.542

Age in years. Abbreviations: R^2^, R-squared; RMSE, root mean squared error; AIC, Akaike information criterion; BIC, Bayesian information criterion; LR-test, likelihood ratio test; mRS, modified Rankin Scale; 6MWT, six-minute walk test.

Table 4 shows that 6MWT added statistically significant value to the prediction of VO_2peak_ in model B (p<0.001). Further, there was a significant interaction term between age and sex (p = 0.040). The mean decline in VO_2peak_ per year was 0.23 ml/kg/min less (95% CI: 0.01 to 0.46) for females than males.

**Table 4:** Results from multiple regression analyses with VO_2peak_ [ml/kg/min] as dependent variable and predictors as listed in each model.

		VO_2peak_ [ml/kg/min]		
*Model A*		B	95% CI	P
Age, years		-0.50	-0.64 to -0.36	< 0.001
Sex, female vs male (at mean age*)		-4.25	-6.89 to -1.61	0.002
Age * sex, female vs male		0.30	0.05 to 0.56	0.019
mRS score				
	mRS 2 vs 1	-4.63	-7.67 to -1.60	0.003
	mRS 3 vs 1	-3.35	-7.57 to 0.87	0.118
***Model B***				
Age, years		-0.35	-0.49 to -0.21	< 0.001
Sex, female vs male (at mean age*)		-1.91	-4.39 to -0.39	0.129
Age * sex, female vs male		0.23	0.01 to 0.46	0.040
mRS score				
	mRS 2 vs 1	-0.73	-3.78 to 2.33	0.638
	mRS 3 vs 1	4.18	-0.51 to 8.88	0.080
6MWT, [m]		0.03	0.02 to 0.05	< 0.001
***Model C***				
Age, years		-0.38	-0.52 to -0.23	< 0.001
Sex, female vs male (at mean age*)		-2.03	-4.72 to -0.66	0.138
Age * sex, female vs male		0.25	0.02 to 0.49	0.036
mRS score				
	mRS 2 vs 1	-2.06	-5.16 to 1.04	0.190
	mRS 3 vs 1	2.64	-2.30 to 7.59	0.291
Max gait speed, [m/s]		7.20	3.56 to 10.85	< 0.001
***Model D***				
Age, years		-0.34	-0.47 to -0.20	< 0.001
Sex, female vs male (at mean age*)		-1.57	-4.13 to 0.99	0.227
Age * sex, female vs male		0.23	0.01 to 0.45	0.045
mRS score				
	mRS 2 vs 1	-0.53	-3.61 to 2.54	0.730
	mRS 3 vs 1	4.90	0.02 to 9.77	0.049
6MWT, [m]		0.03	0.01 to 0.05	0.001
Max gait speed, [m/s]		2.41	-2.09 to 6.91	0.289

Data from a total of 91 participants are presented. Age is mean-centered. Abbreviations: VO_2peak,_ peak oxygen consumption; mRS, modified Rankin Scale; 6MWT, six-minute walk test; B, unstandardized regression coefficient; CI, confidence interval; P, P-value.

*At mean age: The coefficient for the sex difference vary by age due to interaction. The value at the mean age is given.

**Fig 3:**
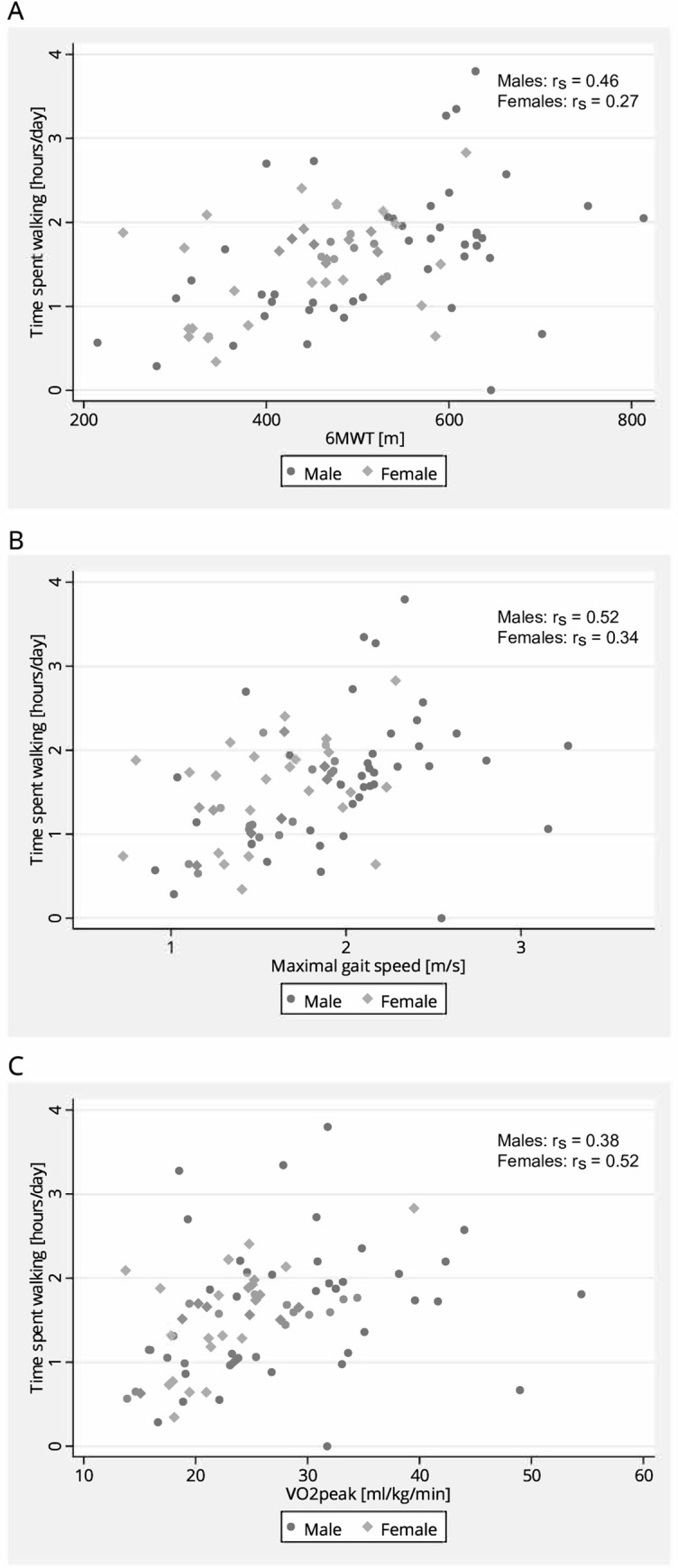
Relationship between time spent walking, functional walk tests and cardiorespiratory fitness. Scatterplots illustrating the relationship between time spent walking [hours/day], and (A) 6-minute walk test (6MWT) [m], (B) maximal gait speed [m/s], and (C) VO_2peak_ [ml/kg/min] in chronic stroke. Abbreviation: r_s_ = Spearman correlation coefficient.

Average time spent walking [hours/day] was found to increase by increased 6MWT, maximal gait speed and VO_2peak_ (p≤0.016), (Table 5). There were no statistically significant interaction effects between sex and the functional walk tests, or sex and VO_2peak_. Fig 3 illustrates the associations between 6MWT, maximal gait speed, and VO_2peak_ and time spent walking.

**Table 5:** Results from three multiple linear regression analyses with walking [hours/day] as dependent variable and independent variables as listed in each regression analyses.

		Walking [hours/day]		
		B	95% CI	P
***6MWT [m]***		0.003	0.001 to 0.004	0.001
Age, years		-0.003	-0.019 to 0.014	0.749
Sex, female vs male (at mean age*)		0.074	-0.235 to 0.384	0.634
mRS score				
	mRS2 vs 1	0.253	-0.143 to 0.649	0.208
	mRS 3 vs 1	-0.001	-0.650 to 0.649	0.998
***Max gait speed [m/s]***		0.560	0.213 to 0.906	0.002
Age, years		-0.006	-0.022 to 0.009	0.421
Sex, female vs male (at mean age*)		0.098	-0.220 to 0.415	0.543
mRS score				
	mRS 2 vs 1	0.177	-0.214 to 0.567	0.371
	mRS 3 vs 1	-0.128	-0.764 to 0.508	0.690
***VO***_***2peak***_ ***[ml/kg/min]***		0.030	0.004 to 0.055	0.023
Age, years		-0.002	-0.021 to 0.017	0.820
Sex, female vs male (at mean age*)		0.063	-0.266 to 0.391	0.706
mRS score				
	mRS 2 vs 1	0.164	-0.241 to 0.568	0.423
	mRS 3 vs 1	-0.254	-0.898 to 0.390	0.435

Data from a total of 84 participants are presented. Age is mean-centred. Abbreviations: 6MWT, six-minute walk test; mRS, modified Rankin Scale; VO_2peak,_ peak oxygen consumption; B, unstandardized regression coefficient; CI, confidence interval; P, P-value. Non-significant interaction terms between age and sex were found in all three regression models and omitted from the final analyses.

*At mean age: The coefficient for the sex difference vary by age due to interaction. The value at the mean age is given.

## Discussion

The primary objective of this study was to investigate whether functional walk tests assessed in the subacute phase after stroke added value in predicting VO_2peak_ in chronic stroke, in addition to age, sex, and functional dependency. This is the first study to give evidence that the 6MWT performed three months after stroke onset added value to the prediction of VO_2peak_ in the chronic phase after stroke. Secondary objectives were to investigate the associations between daily physical activity and functional walk tests, and with VO_2peak_ in chronic stroke. The results showed statistically, but not clinically, significant associations.

To our knowledge, similar prediction models as presented in our study have not previously been published within this patient group. In a cross-sectional study by Harmsen et al., however, they reported an explained variance of VO_2peak_ by 67% when 6MWT was corrected for age and sex in individuals mildly affected with aneurysmal subarachnoid hemorrhage [30]. They concluded that 6MWT can be used to predict mean VO_2peak_ at an aggregated group level [30]. Our study found an only slightly less explained variance of VO_2peak_ by 63.8% (model B) and was further based on a prospective design with a long-term follow-up period, including easily available predictors. However, our results provided a standard deviation for the residuals (i.e., RMSE) of 5.07 ml/kg/min of mean VO_2peak_ (model B), indicating an error too large to accurately predict VO_2peak_ in individual patients. This is in line with others suggesting similar values to have poor prediction accuracy for clinical purposes in an individual level [30, 31]. Predictions of mean VO_2peak_ levels in chronic stroke may provide a reasonable reflection of CRF at a reduced risk and with simpler methods. Such information allows clinicians to choose the most appropriate treatments in patients at risk of low-CRF levels and researchers to identify groups of people eligible for experimental interventions targeting increased cardiorespiratory responses.

Previous research have shown the relationship between walking capacity and VO_2peak_ after stroke to be inconsistent with correlation coefficients to range from 0.29 to 0.74 [11]. However, most studies have been limited to bivariate analyses, which enables only restricted insight into determinants influencing this relationship [11]. Patterson et al., however, have shown that VO_2peak_ explained almost half of the variance (adjusted R^2^ = 48%) in walking distance assessed by 6MWT, and that this accounted for people with milder deficits following stroke, as the association was stronger for those who walked more quickly [32]. The values of the adjusted R^2^ from the reference model and models B and C in our study, indicate that maximal gait speed contributes less to the prediction of VO_2peak_ than 6MWT. Furthermore, maximal gait speed did not contribute significantly in the prediction of VO_2peak_ when added in combination with 6MWT (model D), although both were statistically significant when included one by one. The possible interpretation of this might be the strong multicollinearity between the two functional walk tests (r = 0.86), as both gait speed and distance partly measure the same domains in patients with stroke [33]. Still, with 6MWT remaining statistically significant when combined with maximal gait speed, this strengthen the argument that 6MWT appears to be the strongest predictor of the two functional walk tests. There are indications that gait speed is primarily dependent on neuromotor control and lower body muscle strength rather than CRF [33, 34]. Others speculate whether a short, fast walk, such as required in assessing maximal gait speed, would engage anaerobic metabolism, while a longer walk, such as required by the 6MWT, would engage aerobic metabolism, the latter closer related to VO_2peak_ [35].

Secondary results show statistically significant associations between average time spent walking per day and 6MWT, maximal gait speed, and VO_2peak_ in the chronic stage after stroke. However, the effect sizes indicate that the associations are not reflecting impact on clinical practice. A possible interpretation may be that functional capacity measured in the clinic does not necessarily reflect real-world performance. Rand et al. indicated similar findings in their study, in which participants demonstrated sufficient ability to walk in the laboratory yet revealed low levels of physical activity at home [14]. Our results showed that several of the participants demonstrated an ability to cover a considerable walking distance during the 6MWT, walked with a high maximal gait speed, and demonstrated high values of VO_2peak_. Despite this, there were varying degrees to which this potential was reflected in equivalent high levels of physical activity in their everyday lives. Clinicians should be aware that even individuals with mild motor impairment may present a lower amount of daily physical activity than expected [14]. Hence, this may be an incentive for encouraging and motivating apparently mildly affected individuals to engage in sports, household tasks, and other physical activities in their everyday lives. However, methodological issues may also explain the lack of stronger associations, as the accelerometers did not measure intensity levels during time spent walking. Identifying levels of intensity during daily physical activity would probably show stronger associations between our variables, as higher-intensity levels of daily activities induce an effect on functional capacity [5, 36].

### Study limitations

The number of participants eligible for CPET determined the sample size for these analyses, and most participants who were excluded suffered serious heart disease or other stroke-related impairments contraindicating participation. Hence, selection bias probably occurred toward individuals less affected by stroke, and the results are not generalizable to individuals severely affected by stroke. However, the study sample is fairly representative of the Norwegian stroke population, as the median score of NIHSS is < 3 in acute stroke [37]. Further, the proportion of women and men in our study was similar to the distribution by sex among the Norwegian stroke population in the same age group [37], and more women were included than previously reported in similar studies [11].

Altogether, 15 participants were ineligible for CPET due to very poor walking ability; a bicycle protocol instead may have allowed some of these individuals to participate. However, bicycle protocols risk artificially low levels of VO_2peak_, having shown consistent reports of lower VO_2peak_ values compared to treadmill testing [38]. A limitation of our test procedure was the lack of reporting participants’ uses of medications, in particular, beta-blockers, as recommended by guidelines [39]. A consequence of beta-blockers may be a reduction in VO_2peak_ due to their effect of limiting maximum heart rate and consequently diminished cardiac output [40].

Although participants in our study were included three months post-stroke, after most spontaneous recovery and early rehabilitation has occurred [41], different factors may have affected both walking capacity and CRF during the following months. Still, a prospective design with an 18-month follow-up period should be regarded as a strength compared to several previous cross-sectional analyses within this field [42].

## Conclusions

In conclusion, the present study showed that 6MWT performed in the subacute phase after stroke, added significant value in predicting VO_2peak_ in the chronic phase after stroke, in addition to age, sex and functional dependency after stroke. Hence, this model can be used to predict mean VO_2peak_ in the chronic phase after stroke in mildly affected community-dwelling people. This may facilitate clinical decisions and be useful for research purposes by providing information needed to select and further develop appropriate interventions for groups of people at risk of low CRF levels after stroke. However, the residual standard deviations were too large to accurately predict VO_2peak_ at an individual level. Future studies are necessary to validate the prediction model in various stages after stroke and in patients moderately and severely affected by stroke.
